# Haemostatic agents on the shear bond strength of self-adhesive resin

**DOI:** 10.4317/jced.52284

**Published:** 2015-07-01

**Authors:** Akansha Anil, Anand Sekhar, Manuel S. Thomas, Kishor Ginjupalli

**Affiliations:** 1 Former Under Graduate student, Manipal College of Dental Sciences, Manipal University, Mangalore- 575001; 2 Associate Professor, MDS, Dept. of Conservative Dentistry and Endododntics, Manipal College of Dental Sciences, Manipal University, Mangalore- 575001; 3 MSc, PhD, Dept. of Dental Materials, Manipal College of Dental Sciences, Manipal University, Manipal- 576104

## Abstract

**Background:**

Dentin surface contaminated with haemostatic agents can interfere with the bonding of self-adhesive resin cement. Therefore the purpose of this study was to evaluate the effect of various haemostatic agents such as Aluminium chloride, Ferric sulphate and Tannic acid on the shear bond strength of self-adhesive resin luting agent.

**Material and Methods:**

The buccal surfaces of extracted premolars were flattened to expose the dentine. The teeth were then randomly divided into four groups. In Group I Aluminium Chloride was applied on the flattened dentinal surface, in Group II Ferric Sulphate was applied to exposed dentin surface, in Group III tannic acid was applied on to the dentinal surface, and the control group, i.e. Group IV was rinsed with saline. After the surface treatment, all the teeth were air dried. Then a predetermined dimension of RelyX™ U200 self-adhesive resin cement was bonded to the pretreated dentin surfaces. The samples were then stored under 370C in distilled water for 24 hours under 100 % humidity. Following this each sample was tested for shear bond strength with an Instron testing machine at a crosshead speed of 1mm/min.

**Results:**

There was significant difference in the shear bond strength of control and tannic acid contaminated group (*p*<0.05), whereas there was no significant differences between the shear bond strength between control and aluminium chloride and ferric sulphate groups (*p*>0.05).

**Conclusions:**

The usage of haemostatic agent can negatively affect the bond strength of self-adhesive resin cement (Rely X) on to the dentin surface. As per the study Tannic acid significantly weakened the bond between the self-adhesive resin and dentin.

** Key words:**Aluminium chloride, Ferric sulphate, haemostatic agent, self-adhesive resin cement, shear bond strength, Tannic acid.

## Introduction

Self-adhesive resin cements (SARC) were developed to make luting procedures simple, reduce treatment time and minimize technique sensitivity related with multiple step adhesive procedures (1). This material is applied directly on the dentin surface, without the need for any dentin pretreatment. Incorporation of the acidic monomers into the smear layer as well as chemical interaction between phosphoric acid monomers and the hydroxyapatite of the dental hard tissues have been postulated as reasons for the self-adhesive nature of these resin cements (2,3). Rely X™ U200 is an example of one such self-adherent universal cement. This SARC can be used in the cementation of any indirect restorations made of metal alloys, or all-ceramics or composites (4).

With the greater demand and use of aesthetic restorations, control of moisture has become an important concern. Dental adhesives and composites resins are highly susceptible to moisture contamination. Moisture such as gingival crevicular fluid, blood and saliva can affect the quality of the bond, leading to microleakage at the interface. This may ultimately result in postoperative sensitivity, recurrent caries, and/ or the loss of restoration. Hence, proper isolation and moisture control is crucial during adhesive procedures (5). Previous studies have shown that blood contamination on the dentin surface can cause a marked reduction in the bond strength at dentin-resin interface (6,7). So to control the bleeding, haemostatic agents like ferric sulphate, aluminium chloride and tannic acid can be used. However, some of these haemostatic agents by themselves may decrease the shear bond strength of dental adhesives. So in the wake of this, our study focuses on knowing the effect of different haemostatic agents on the bond strength of self-adhesive resin cement.

## Material and Methods

-Sample selection: Forty extracted human premolars were selected based on the inclusion criteria that there was no evidence of caries, no restorations and lack of any cracks or fractures on the crown. These sample stored in distilled water at room temperature.

**Figure 1:**
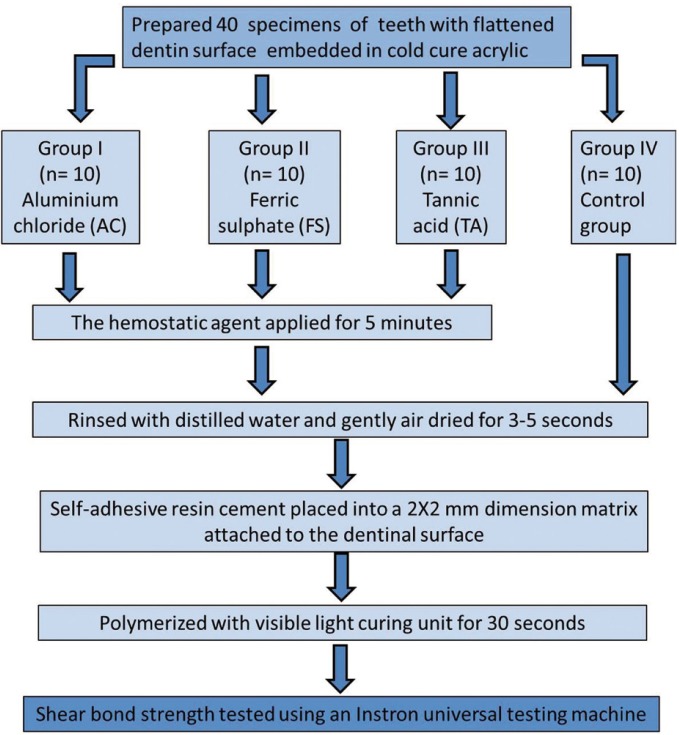
Schematic diagram representing the experimental procedure and testing groups in this current study.

-Sample preparation: The selected teeth were then embedded in auto-polymerizing acrylic resin such that 2mm of root surface along with crown was exposed. The buccal surfaces of the specimens were flattened to expose the superficial dentine using a diamond abrasive under water coolant. The surface was then finished using a 600 grit silicon carbide abrasive. The teeth were then rinsed with distilled water to remove any debris. Following this the specimens were randomly divided into four groups (n= 10) based on the hemostatic agent used for surface treatment (Fig. 1). The details of the materials used in this study are shown in  Table 1.

**Table 1:**
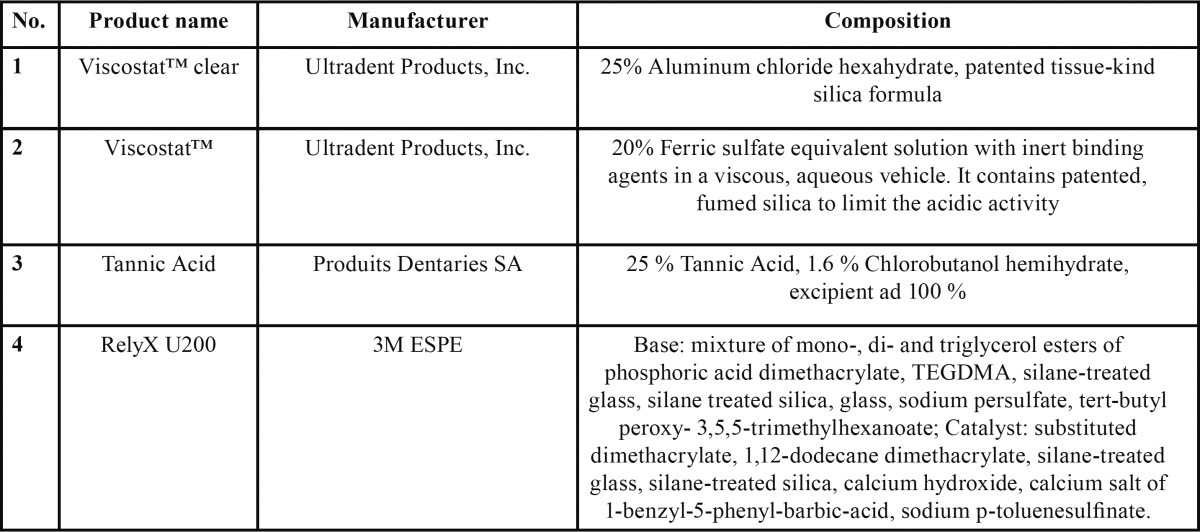
Materials used in the study.

-Grouping: The various groups are as follows

Group I- Aluminium chloride (AC) 25 % (Viscostat™ clear; Ultradent Product Inc., Utah) was applied on to dentin surface for 5 minutes, surface rinsed with distilled water and gently air dried for 3-5 seconds.

Group II- Ferric sulphate (FS) 15% (Viscostat™; Ultradent Product Inc., Utah) was applied for applied for 5 minutes, surface rinsed with distilled water and air dried for 3-5 seconds.

Group III- Tannic acid (TA) 25% (Produits Dentaries SA, Vevey, Switzerland) was applied for applied for 5 minutes, surface rinsed with distilled water and air dried for 3-5 seconds.

Group IV- In the control group, the uncontaminated dentin surface was rinsed with distilled water and air dried for 3-5 seconds.

The SARC (Rely X-U200, 3M, ESPE, St. Paul, MI) was manipulated as per the manufacturers’ instructions. The material was filled into a plastic matrix of 2 mm height and 2 mm internal diameter attached to the middle of the treated dentinal surface. The specimens were polymerized using visible light curing unit (Elipar 2500, 3M ESPE, St. Paul, MN) for 30 seconds. The specimens were then evaluated for the strength of bonding between the SAR to the treated dentin surface.

-Storage of the samples: After bonding Rely X to the dentinal surface, the specimens were stored under 370C in distilled water for 24 hours under 100% humidity.

**Figure 2:**
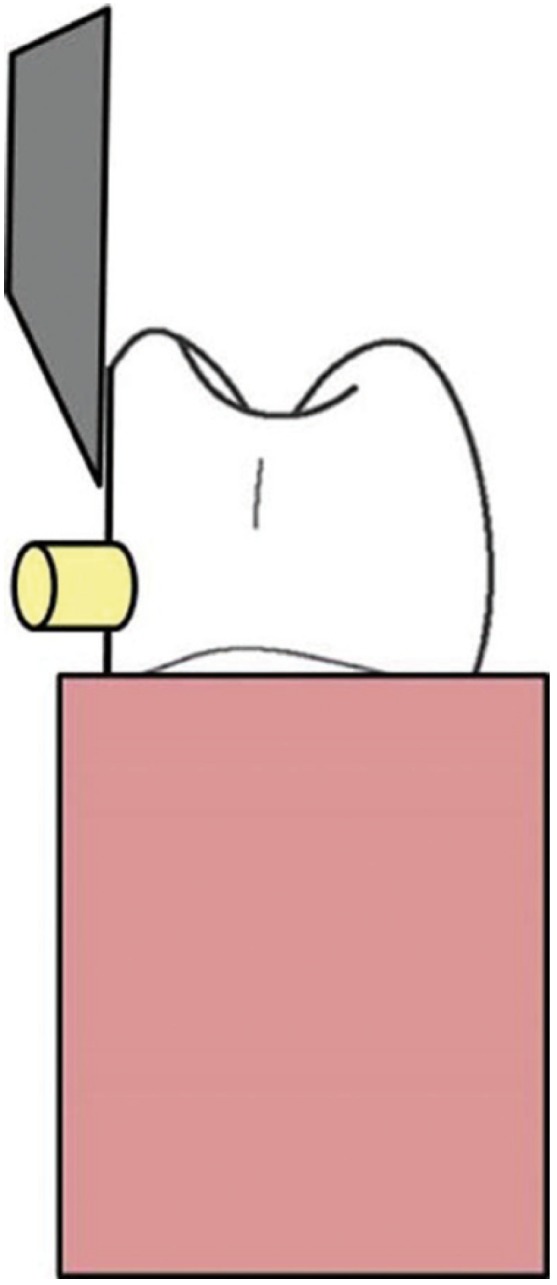
Schematic diagram representing the test assembly for determining shear bond strength.

-Measurement of shear bond strength: Samples were then placed into a positioning jig and tested in shear with an Instron Testing Machine (Instron Corporation, Canton, MA) using a crosshead speed of 1 mm per minute (Fig. 2). The shear bond strengths of the samples were calculated and expressed in MPa.

-Determination of the mode failure: Fracture analysis of the bonded dentinal surface was performed under stereomicroscope of 20 X magnification (Reichert, Stereo Star Zoom-570). Fractures were classified as adhesive (more than 3/4 of the failure was at tooth and restorative interface), cohesive (more than 3/4 of the failure was within the luting agent), or a mixed failure.

-Statistical analysis: The collected data was presented as mean and standard deviation. One –way ANOVA test was used to compare the groups and multiple comparisons were performed using Tukey HSD method. For the analysis the level of significant was set at *p*<0.05.

## Results

**Figure 3:**
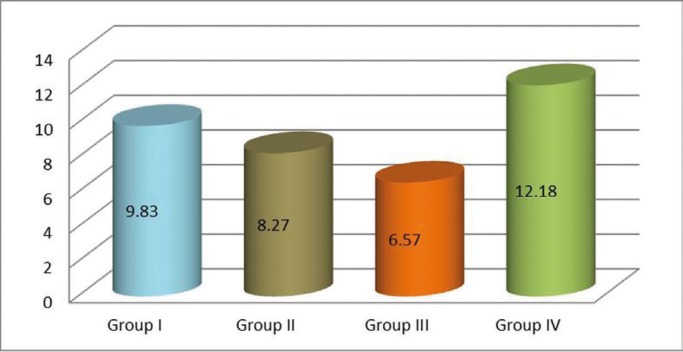
Graph representing the mean bond strength (in MPa) of self-adhesive resin cements in various groups.

The highest shear bond strength (SBS) of self-adhesive resin cement (SARC) was observed in the control group [12.18±3.90] and the least was observed in Group III, i.e. the Tannic acid group [6.57±4.15] (Fig. 3). Both these groups showed statistically significant difference (*p*<0.5) ( Table 2). Even though Group II (Aluminium chloride) [9.83±1.50] and Group III (Ferric sulphate) [8.27±4.36] showed reduced SBS as compared to the control, there was no statistically significant difference. Irrespective of the group all the samples showed adhesive failure ( Table 2).

**Table 2:**
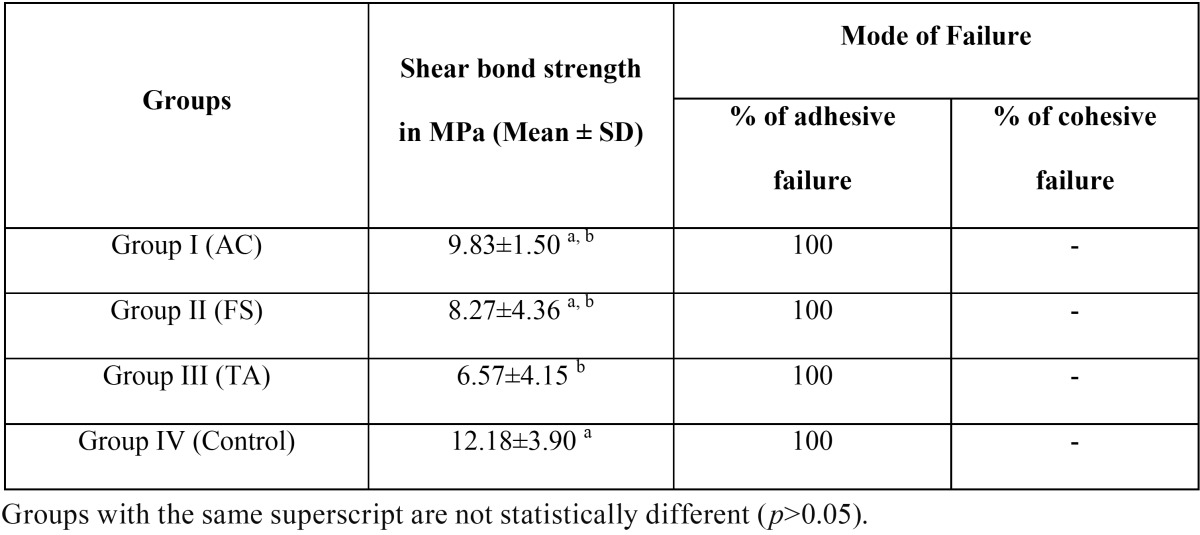
Mean of the bond strength values (MPa) and respective standard deviation(± SD) and the percentage of failure mode of self-adhesive resin cements bonded to tooth surface contaminated with various hemostatic agents.

## Discussion

In the current study, macro-shear bond-strength method was used for testing the bonding efficiency of SARC to hemostatic contaminated dentin surface. It is the most commonly used method for bond-strength testing. It is highly popular as it is the most easy and fastest method, because no further specimen processing is required after the bonding procedure (8). In the present study, efforts were taken to reduce the influence of confounding factors such as the tooth surface variability by standardizing the depth of the flat dentine surface as well as the thickness of smear layer (9). The specimens were finished using a 600- grit silicon carbide paper to produce a thicker and a uniform smear layer on the dentinal surface (10). In the cementation stage of indirect restorations, bleeding mostly occurs around the gingival margin and the clinician applies the haemostatic agent around the gingiva (11). Therefore, SARC was bonded to the cervical third of the flat dentin surface to simulate the clinical situation. Since the purpose of the current study was primarily to determine the effects of haemostatic agents alone on bond strength of resin cement to dentin, the sample where not contaminated with blood before the application of the test solutions (11). As the purpose of this study was solely to assess the bond strength between contaminated dentin surface and SARC in the absence of any additional stresses, the specimens were not subjected to thermocycling (12).

RelyX U200, the self-adhesive resin cement used in the presented study consists of alkaline fillers and multifunctional phosphoric acid methacrylates, which are responsible for its self-etching and adhesive properties. When compared to other studies (2,13), the SARC in the current study showed poor adhesiveness as demonstrated by adhesive failure of all the test samples. The reason for this could be because of improper adaptation of the highly viscous resin cement on to the dentin due to the passive placement (2,14). Probably, pressure during the placement and curing of SARC on dentin could have resulted in better bond strength (12). The variation in the bond strength values could also be attributed to the method used to dry the dentin surface. SARCs showed the best bonding performance when only a small amount of water remained on the dentin surface (15).

The two probable means by which a material can adhere to the dentinal surface is ether by the formation of resin interdiffusion zone or through direct chemical interaction. It has been demonstrated that the SARC are incapable to demineralize or dissolve the smear layer completely as well as unable to diffuse and decalcify the underlying dentin to form hybrid layer or resin tags (2). This inability of the SARCs to diffuse and demineralize the underlying dentin effectively could be the result of its’ high viscosity and neutralization effect that occurs during the setting because of the chemical reactions involve water release and alkaline filler that may raise the pH level (16). However, study by Gerth *et al.* (3), showed an intense chemical interaction of the self-adhesive cements with calcium from hydroxyapatite. Therefore, due to the limited micromechanical retention, the bonding of SARC to tooth substrate may be more dependent on a chemical interaction between the acidic monomers and the calcium in the hydroxyapatite. Thus a strong bond between dentin and SARC can be achieved only when functional groups in the acidic monomers of SARCs produce an optimal interaction with hydroxyapatite on the dentin surface (17).

It can be reasoned from the current results that the decreased availability of Ca+2 on the dentin surface due to the decalcifying effect of tannic acid could have reduced the chemical bonding of SARC on to the tooth surface. The change in the calcium/phosphorus ratio of the dentin surface can alter both the chemistry and morphology of the dentin surface in a manner that can compromise the bond strength of SARCs (13). Removal of smear layer and exposure of the hydrophobic component of dentin can also be assumed as the reason for reduction of bond strength in the tannic acid contamination group. Rely X Unicem has been considered as hydrophobic (15). Hydrophilic surface created could impede the interaction of more hydrophobic materials such as the BisGMA monomer present in RelyX, thus reducing the overall wettability of the cement (1).

Previous studies have shown than Aluminum chloride (AC) and ferric sulphate (FS) dentin contamination can significantly lowered the bond strength of self-etch adhesive compared to normal dentin (18-20). However in the current study, thought the AC and FS contamination groups showed lowering in the bond strength, it was not statistically significant. This could have been attributed to the difference in the methodology of the sample preparation, bonding and testing methodology. The patented fumed silica within these hemostatic agents could have limited its acidic activity. Nevertheless, the discoloration potential combined with the reduction in bonding of ferric sulphate based hemostatic agents should caution the clinicians in using these agents with esthetic restoration especially cemented with SARC (21). Further studies evaluating the effectiveness of various decontamination techniques after application of hemostatic agents should be investigated.

## Conclusions

Considering the limitation of an *in vitro* methodology, the results of the present study showed that the usage of haemostatic agent can negatively affect the shear bond strength of a self-cure adhesive resin cement to dentin. Tannic acid containing hemostatic agents needs to be used with caution as it has shown significant reduction in the adhesive properties of SARCs. Although Aluminium chloride and Ferric sulphate containing hemostatic agents showed lowering in the bond strength of SARCs, it was not statistically significant. In cases where the margins of esthetic tooth preparations are subgingival and gingival bleeding needs to be controlled, then the ginigival retraction fluid of choice would be Aluminum chloride containing hemostatic agents because of its fewer drawbacks.
